# Supplementary material for: Interplay between Telecommunications and Face-to-Face Interactions: A Study Using Mobile Phone Data
Source: PLoS One. 2011 Jul 13;6(7):e20814. doi: 10.1371/journal.pone.0020814 (PMC3135588; doi:10.1371/journal.pone.0020814)
Supplement: Text S1 — Dataset. (PDF) [file pone.0020814.s001.pdf]

S1 Dataset

The dataset corresponds to approximately 435 million Call Detail Records (CDR) collected from a telecom operator in Portugal. To safeguard personal privacy, individual phone numbers were anonymized by the operator before leaving their storage facilities, and where identified with a security ID (hash code). Each call is recorded with a:

- timestamp,
- originating user id,
- terminating user id,
- call duration,
- originating user tower id,
- terminating user tower id.

Each user that makes or receives a call is located through its connected cellphone tower (at the time the call was initiated). There are 6509 cell towers (tower ids), each serving on average an area of approximately 14 km^2, which reduces to $0.13 km^2$ in urban areas such as Lisbon.

The dataset covers 406 non consecutive days, between 2006 and 2007.

In accordance with [1] we reduced the dataset to only consider reciprocal communications between users. By grouping together all calls made between two users, we derived a network of 1.8 million nodes (1822756 precisely) and 11M edges (11367729 precisely). The distributions of node degree, number of calls, and total duration of calls are reported in Figure 1.

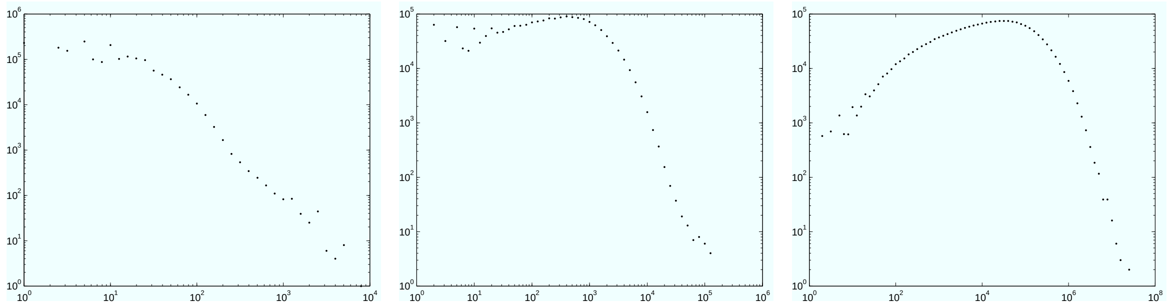

(a) Number of contacts (b) Number of calls (c) Total call time

Figure 1. Degree distributions of node degree, number of calls, and total duration of calls

With the interactions network and locations, we were able to evaluate where two people are at the time of the call. Figure 2 shows the cumulative distribution of distances for all users.

Starting with the full dataset of 435 million CDRs, we subsampled it to discard users that had insufficient location information. We ranked users by the number of calls they made or received, and further subsampled the dataset only considering information for the first 100,000 users. This corresponds to at least 1846 personal records in the whole observation period (4.5 records per day). This subset (D1) contains over 1 million reciprocal communications between the selected users.

We then estimated the users' home location and considered 10,000 random pairs of communicating users, sampled at different home distances to have the same home distances distribution found in D1 (see Text S2). We denoted the second subset as D2 and used it in cases where computational complexity limits the use of a larger set.

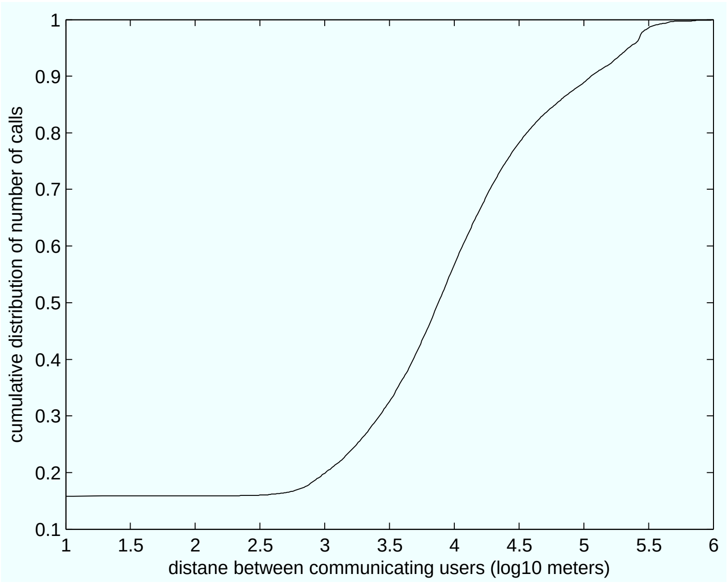

Figure 2. Cumulative distributions of calls as a function of the distance between users

Some limitations must be addressed when considering these datasets. A crucial parameter to take into account is the localization accuracy, which limits the minimum size of the regions that can be considered. In our case, the location information is extrapolated from the location of the cellphone tower the user is connected to, and assigned as the centroid of the cell tower coverage area, which is in turn determined through Voronoi tessellation of the space. Other factors that may affect the statistical results include: 1) the market share of the mobile phone operator from which the dataset is obtained, 2) the potential non-randomness of the cellphone users (e.g. teenagers), 3) calling plans which can influence the number of samples acquired over varying temporal ranges, 4) the number of devices that each person carries. Nonetheless, the study performed on the spatial distribution of cellphone users confirm that the mobile phone data represent a reasonable proxy for the human population.

References

1. Onnela J, Saramaki J, Hyvonen J, Szabo G, Lazer D, et al. (2007) Structure and tie strengths in mobile communication networks. Proceedings of the National Academy of Sciences 104: 7332.
