# Supplementary material for: Interplay between Telecommunications and Face-to-Face Interactions: A Study Using Mobile Phone Data
Source: PLoS One. 2011 Jul 13;6(7):e20814. doi: 10.1371/journal.pone.0020814 (PMC3135588; doi:10.1371/journal.pone.0020814)
Supplement: Text S2 — Home and work location determination. (PDF) [file pone.0020814.s002.pdf]

S2 Home and work location determination

We considered all calls made between 10pm and 7am, and selected the most frequent tower id each user is at call time, thus defining it as the user's home tower. Each tower is assigned an approximate reception area from the Voronoi diagram generated from the tower locations.

By assigning each tower to the municipality where its coverage area is most extensive, we assigned each user to a residence municipality. We compared the number of users to the municipality population extracted from the Instituto Nacional de Estatstica 2001 Census [1] (depicted in Fig. 1), and found linear correlation with share of about 5.5% for municipalities with at least 10000 people (see Fig. 2). The mismatch found for smaller municipalities could be attributed to the approximate manner in which the assignment between towers and municipalities took place.

By considering the home locations, we evaluated the distribution of distances between users' home locations. We found a distribution centered around 10 km, and with most distances concentrated between 1 and 100 km.

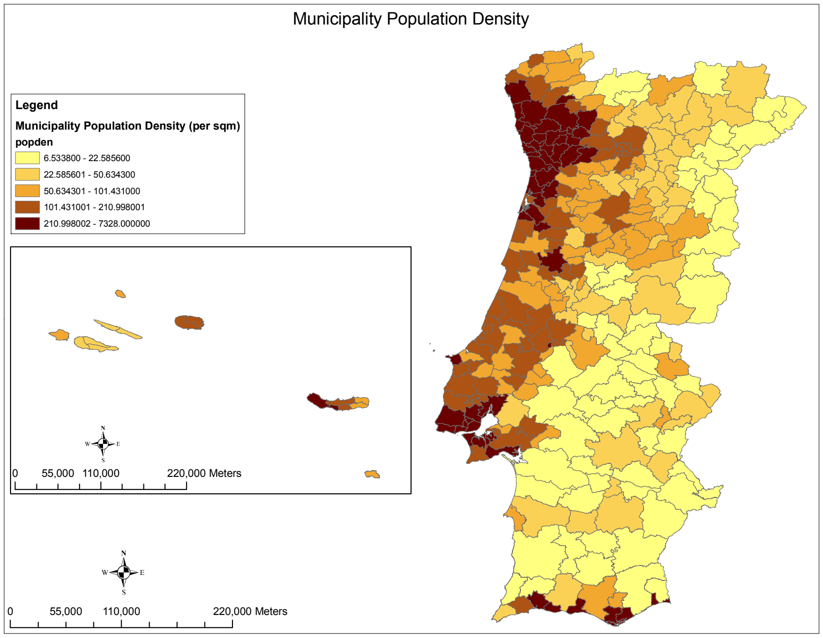

Figure 1. Portugal's Municipality population density.

Following a similar approach, we also considered all calls made between 9am and 5pm, and selected the most frequent tower id each user had at call time, defining it as the user's work tower.

References

1. Instituto nacional de estatística - 2001 census. URL `http://www.ine.pt`.

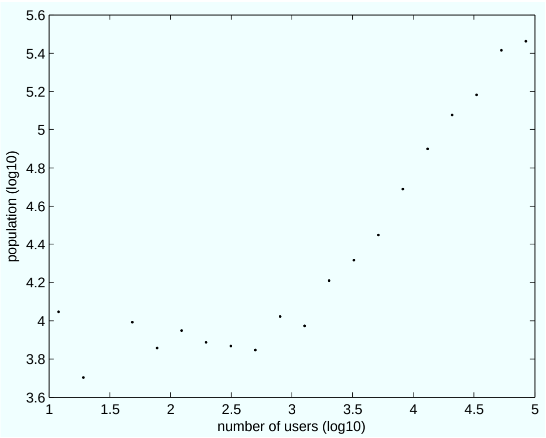

(a) Comparison with Municipality population.

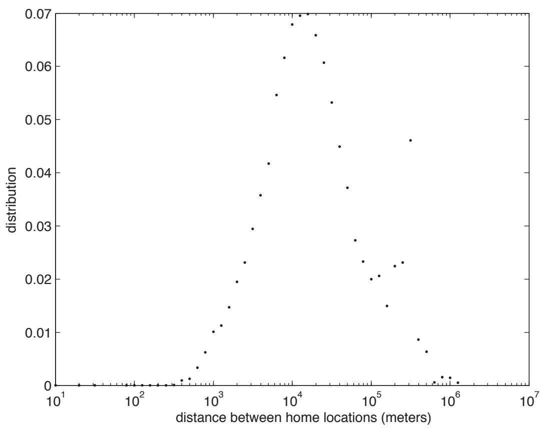

(b) Distribution of distance between users' home locations.

Figure 2. Statistics on the estimated home locations.
