# Supplementary material for: Interplay between Telecommunications and Face-to-Face Interactions: A Study Using Mobile Phone Data
Source: PLoS One. 2011 Jul 13;6(7):e20814. doi: 10.1371/journal.pone.0020814 (PMC3135588; doi:10.1371/journal.pone.0020814)
Supplement: Text S3 — Co-location places, geography and communication strength. (PDF) [file pone.0020814.s003.pdf]

S3 Co-location places, geography and communication strength

We analyzed the features of co-location places and compared it with geographical and communication differences between users. We defined $d_1(l)$ and $d_2(l)$ as the distances traveled by two users at every co-location event $l = 1, \ldots, m$, and computed three measures of comparison:

1. The median ratio between the shortest and longest distance at co-location time:

$$r_d = median_l \frac{min\{d_1(l), d_2(l)\}}{max\{d_1(l), d_2(l)\}}.$$

2. The fraction of times user 1 travels less than the peer:

$$r_{t1} = 1/m \sum_{l=1}^{m} g(d_2(l) - d_1(l)),$$

where:

$$g(x) = \begin{cases} 1 & if\ x > 0 \\ 0 & if\ x = 0 \end{cases}.$$

3. The fraction of times one of the users travels less than the peer:

$$r_t = min\{r_{t1}, r_{t2}\}.$$

We found that r_d decreases from 0.35 to 0.05 as homes distance increases (see Figure 1(a)). This reveals that the greater the distance between users, the more likely they are to meet in a place that is closer to one's home location. Ratios r_l were very small (see Figure 1(b)), ranging from 0.1 to 0.05 as homes distance increases, showing that people tend to co-locate most of the time in a place that is closer to the same individual.

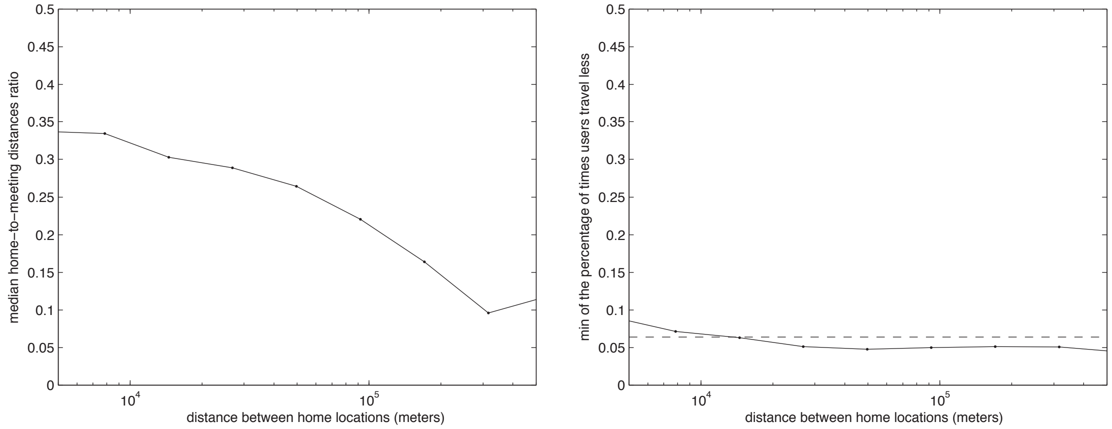

(a) r_d (b) r_l

Figure 1. Statistics on places where people co-locate as function of how far they live (D2 subset).

We looked at other geographical features that can influence the decision to travel. We evaluated whether living in a high or low density area influences the decision to travel. For each user, we computed

the home municipality's population density (pd), and evaluated the ratio, compared it to r_d and r_{l1}:

$$r_{pd} = \frac{pd_1}{pd_2}.$$

The results in Figures 2(a) and 2(b) show that r_d decreases from 0.3 to 0.05 as the ratio between population densities increases. This demonstrates that the more people live in differently populated areas, the more they will meet close to one of the two homes. No trend is instead visible on r_{l1}, which is around the expected value 0.5.

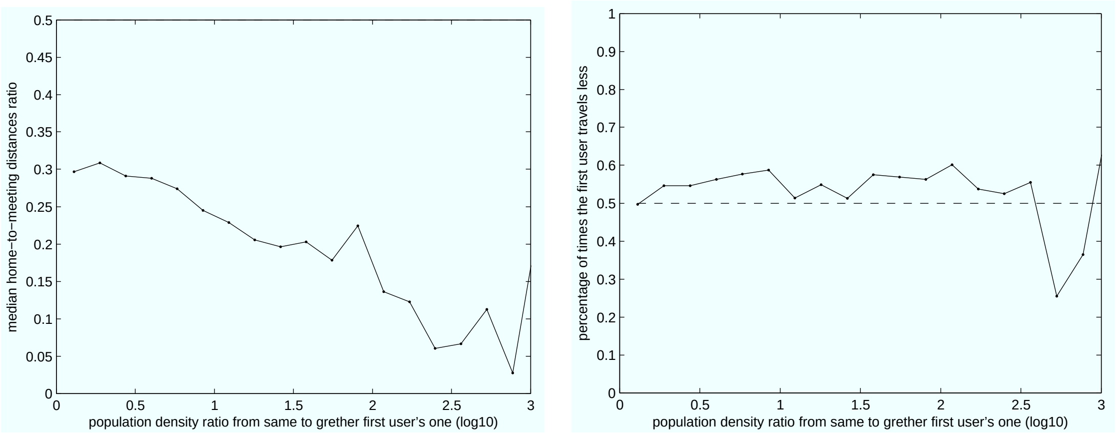

(a) r_d (b) r_{l1}

Figure 2. Statistics on places where people co-locate as function of homes population density (D2 subset).

We looked at how differences in tie strength correlate with co-location distances. We normalized each measure of telecommunication tie weight $w_{i\to j}$ (total call duration) between two users by its expected weight in a randomized network. The normalized weight on the directed tie became $w^n_{i\to j} = \frac{w_{i\to j}}{w^{null}_{i\to j}}$, where

$$w^{null}_{i\to j} = \frac{\sum_{k\in N(i)} w_{i\to k}}{|N(i)|}.$$

This is equivalent to normalizing a node's weight by the average weight on the node's ties. When $w^n_{i\to j}$ is larger than 1, the weight is stronger than in the random null model, and we considered $i \to j$ to be a *strong tie*. Conversely, if it is smaller than 1, the weight is weaker than randomly expected, and we considered $i \to j$ to be a *weak tie*. If it is equal to 1, we considered the tie to be neither weak nor strong. To compare the tie strength of two communicating users, we computed the ratio:

$$r_{ts} = \frac{w^n_{i\to j}}{w^n_{j\to i}} = \frac{w_{i\to j}}{w^{null}_{i\to j}} \cdot \frac{w^{null}_{j\to i}}{w_{j\to i}} = \frac{w^{null}_{j\to i}}{w^{null}_{i\to j}}.$$

The greater the difference in the normalized tie strength between users, the more co-locations happen in places close to one of them (see r_d in Figure 3(a)). Moreover, the stronger the tie of a user compared with a peer, the more times he/she will travel less to reach co-location places (see r_{l1} in Figure 3(b)).

We also looked at the effect of call direction (who calls who) on the distances to co-location places. Defining $dir_1(c)$ and $dir_2(c)$ as the number of calls from user 1 to user 2 and vice-versa, we computed

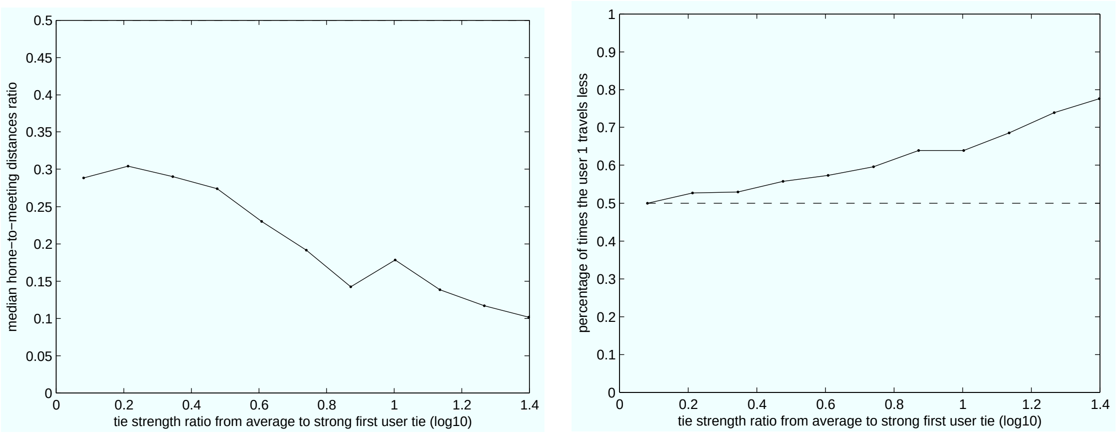

(a) r_d (b) r_{l1}

Figure 3. Statistics on places where people co-locate as function of normalized telecommunication tie strength (D2 subset).

the ratio:

$$r_{dir} = \frac{dir_1 - dir_2}{dir_1 + dir_2},$$

and compared it to r_d and r_{l1}. However, the results in Figures 4(a) and 4(b) do not show clear correlations.

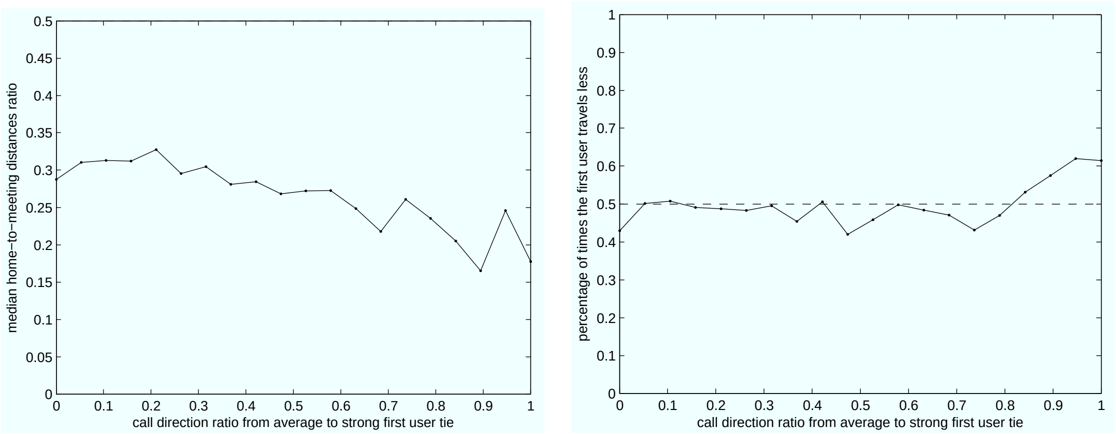

(a) r_d (b) r_{l1}

Figure 4. Statistics on places where people co-locate as function of call direction (D2 subset).
