# Supplementary material for: Interplay between Telecommunications and Face-to-Face Interactions: A Study Using Mobile Phone Data
Source: PLoS One. 2011 Jul 13;6(7):e20814. doi: 10.1371/journal.pone.0020814 (PMC3135588; doi:10.1371/journal.pone.0020814)
Supplement: Text S4 — Statistical analysis. (PDF) [file pone.0020814.s004.pdf]

S4 Statistical analysis

The purpose of this section is to support our findings with rigorous goodness-of-fit analysis. For result in Figure 6, we provide the parametric model that give the best fit of the data with 95% confidence interval:

$$log_{10}\#colocations = a + b \cdot log_{10}\#calls + c \cdot log_{10}distance$$
$$a = -0.0842 \pm 0.0146$$
$$b = 0.6042 \pm 0.0021$$
$$c = -0.0822 \pm 0.0034$$

Using standard curve fitting analysis, we evaluated goodness-of-fit statistics calculating the sum of squares due to error (SSE) $2.6 \cdot 10^4$, the R-square 0.61, adjusted R-square 0.61 and the root mean squared error (RMSE) 0.37.
