# Supplementary material for: Interplay between Telecommunications and Face-to-Face Interactions: A Study Using Mobile Phone Data
Source: PLoS One. 2011 Jul 13;6(7):e20814. doi: 10.1371/journal.pone.0020814 (PMC3135588; doi:10.1371/journal.pone.0020814)
Supplement: Text S5 — Relationship between co-locations and calls over time. (PDF) [file pone.0020814.s005.pdf]

S5 Relationship between co-locations and calls over time

We examined how the relationship between calls and co-locations changes over time, to assess whether there is any temporal changes in the relationship found. To do so, we counted the number of calls and co-locations that each pair of users made each week, and looked at its variation over the course of the monitored weeks. Results are shown in Figure 1. As no clear trend was found in the timeframe examined, we can conclude that both telecommunication (calls) and physical (co-locations) interactions are important to keep social relationships and that one of them does not tend to substitute the other.

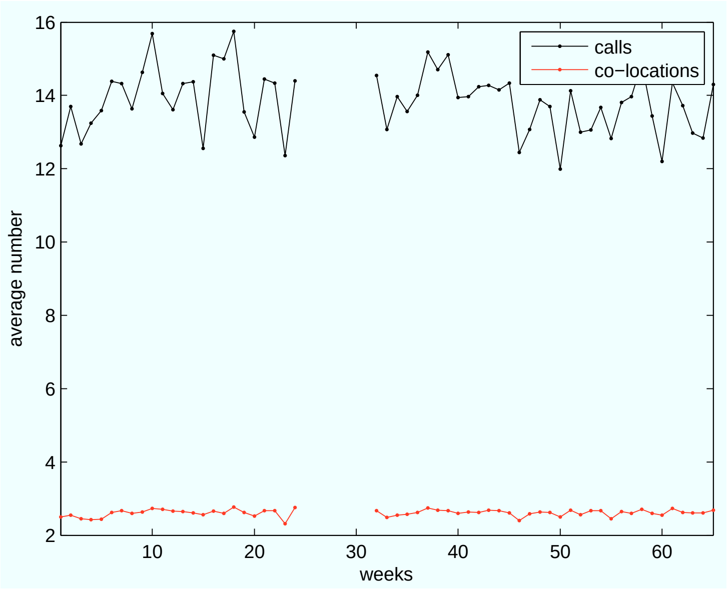

Figure 1. Temporal analysis of calls and co-locations. There are gaps in the dataset between weeks 25 and 31 (D2 subset).
